# Multifocal Targetoid Lesions Due to Disseminated Tuberculosis

**DOI:** 10.4269/ajtmh.25-0494

**Published:** 2026-01-06

**Authors:** Venkatesh Vaithiyam, Aman Agarwal, Sanjeev Sachdeva

**Affiliations:** Department of Gastroenterology, GB Pant Hospital and Associated Maulana Azad Medical College, New Delhi, India

## INTRODUCTION

**Figure 1.**
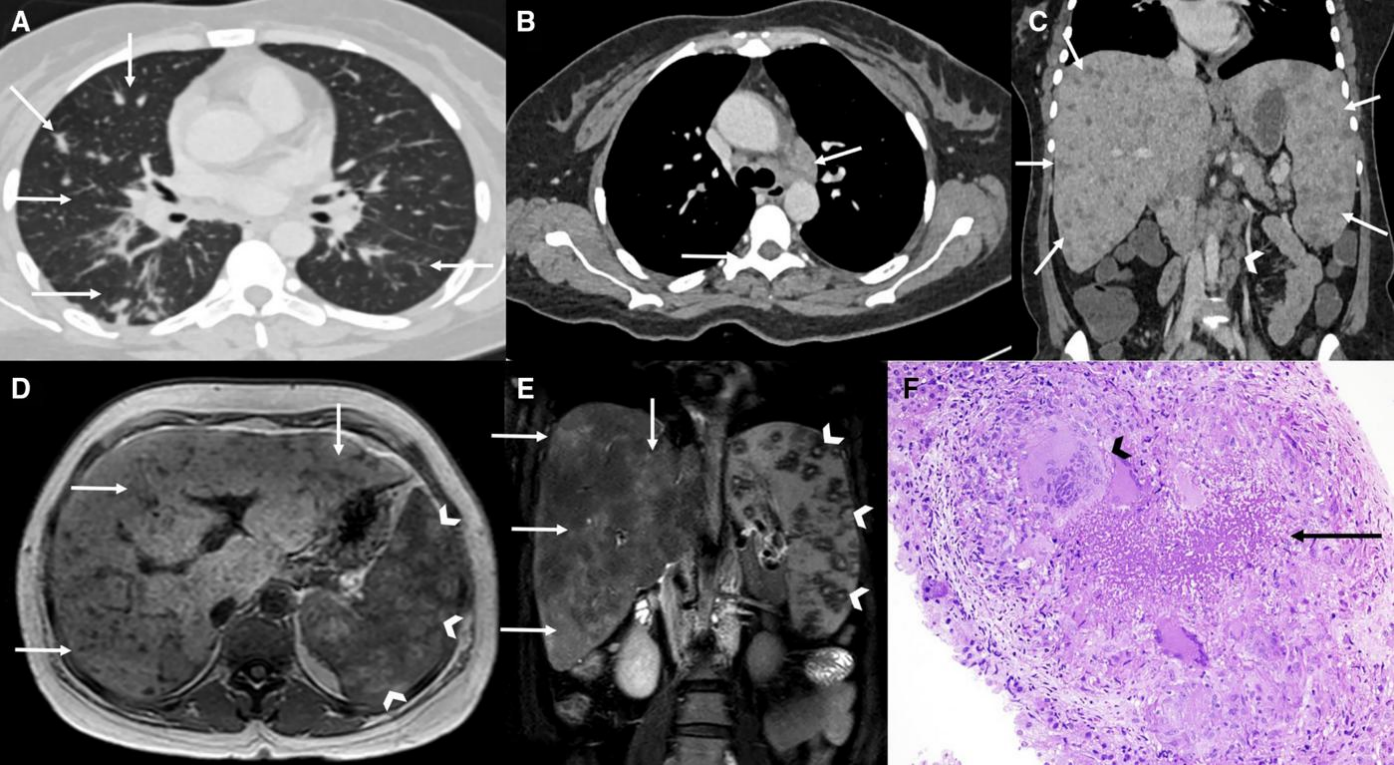
A contrast-enhanced computed tomography (CECT) scan of the chest revealed centrilobular lung nodules, mediastinal lymph nodes (**A** and **B**). A CECT scan of the abdomen revealed retroperitoneal lymphadenopathy and multiple target-like lesions in the liver and spleen (**C**). Magnetic resonance imaging of the abdomen also revealed multiple target-like lesions in the liver and spleen (**D** and **E**). An ultrasound-guided biopsy of the liver lesion revealed multiple large caseating granulomas; however, acid-fast bacilli staining results were negative (**F**).

A 39-year-old woman of low socioeconomic status with no previous comorbidities presented with 5 months of abdominal pain and 2 months of jaundice, weight loss, and anorexia. The intermittent jaundice was not associated with clay-colored stools, pruritus, fever, or an abdominal lump. Her husband had been treated for pulmonary tuberculosis (TB) 2 years previously. A physical examination revealed pallor, mild icterus, and hepatosplenomegaly. Her hemoglobin level was 6.6 g/dL, and her erythrocyte sedimentation rate was 98 mm/hour. A peripheral smear revealed normocytic, normochromic red blood cells with high ferritin levels, decreased total iron binding capacity, and iron levels consistent with anemia of chronic disease. Her serum bilirubin level was 4.1 mg/dL, alanine transaminase level was 18 U/L, and alkaline phosphatase level was 1,060 U/L. The serum angiotensin-converting enzyme level was 98 IU/L, and the human immunodeficiency virus ELISA test result was negative. A contrast-enhanced computed tomography (CECT) scan of the chest revealed centrilobular lung nodules and mediastinal lymph nodes (Figure 1A and B). A CECT scan of the patient’s abdomen revealed retroperitoneal lymphadenopathy and multiple target-like lesions in the liver and spleen (Figure 1C). Magnetic resonance imaging of the abdomen also revealed multiple target-like lesions in the liver and spleen (Figure 1D and E). An ultrasound-guided biopsy of the liver lesion revealed multiple large caseating granulomas; however, staining results for acid-fast bacilli (AFB) were negative (Figure 1F). On the basis of the patient’s history, radiological evidence, and histological findings, a diagnosis of disseminated TB was considered. She was started on antitubercular therapy and exhibited significant clinical improvement at a 3-month follow-up.

Disseminated TB, characterized by hematogenous spread to two or more noncontiguous organs, is a serious mani festation of *Mycobacterium tuberculosis* infection. Hepatic involvement occurs in up to 80–100% of disseminated TB cases, whereas primary hepatic TB is rare.^1^ Clinical jaundice in hepatic TB is uncommon and usually indicates intrahepatic granulomatous inflammation or extrinsic biliary compression caused by tuberculous lymphadenopathy. Splenic TB is also a part of disseminated TB, and it can present as abscess, miliary, nodular, or mixed types analogous to the spectrum seen in hepatic TB.

Imaging can reveal hepatosplenomegaly, hypo- or hyperechogenic masses, tubercular abscesses, biliary strictures, and widespread abdominal lymphadenopathy, the most common associated feature.^2^ Contrast-enhance computed tomography and magnetic resonance imaging of hepatosplenic (micronodular) miliary TB, the most common form, reveal multiple small, ill-defined nodules measuring 0.5–2 mm in the liver and spleen.^3^ On CECT scans, these appear as hypodense lesions with little or no enhancement after contrast administration. The concentric configuration, comprising a central hypointense or necrotic core, a hyperintense rim indicating granulation or inflammatory tissue, and an outer hypointense fibrotic zone, creates a distinctive target-like appearance on contrast-enhanced imaging. Magnetic resonance imaging is more sensitive than CECT in detecting small granulomas, with tuberculoma characteristics varying by lesion stage. In non-caseous granulomas, lesions appear isointense or hypointense on T1-weighted MRI image and hyperintense on T2. In the caseous stage, the necrotic core becomes iso- to hypointense on T1 with a hyperintense peripheral ring, and hypointense on T2. Non-caseous granulomas with liquefied centers show iso- to hypointense T1 signals with a hyperintense peripheral ring and hyperintense T2 signals.^3^^,^^4^

The differential diagnoses for target-like lesions in the liver and spleen include metastases, fungal abscesses, TB, and lymphoma. Less than 10–15% of patients with hepatosplenic TB display a clear target-like pattern, and the frequency may vary depending on the imaging modality and disease stage. Liver biopsy remains a key diagnostic tool, often revealing caseating granulomas. Acid-fast bacilli positivity for hepatosplenic TB varies from 0% to 60%.^5^^,^^6^ Polymerase chain reaction testing and GeneXpert Mycobacterium tuberculosis/Rifampicin (Cepeheid, Sunnyvale, CA) can improve microbiological confirmation.^7^
